# Anterior tibial tendon transfer in idiopathic clubfoot: pull-out vs. other fixations – a systematic review

**DOI:** 10.1186/s12891-024-07621-9

**Published:** 2024-08-12

**Authors:** Andreia Moreira, Luciano Benjamin Ravetti, Douglas Carrapeiro Prina, Monica Paschoal Nogueira

**Affiliations:** 1 Hospital Pedro Hispano, ULS Matosinhos, Portugal, Matosinhos, Portugal; 2 Hospital Universitario Austral, Buenos Aires, Argentina; 3 Hospital do Servidor Publico Estadual de São Paulo, Sao Paulo, SP, Brazil; 4 Núcleo de Apoio à Pesquisa Ortopédica Avançada (NAPOA), Sao Paulo, SP Brazil

**Keywords:** Clubfoot, Tendon transfer, Systematic review

## Abstract

**Purpose:**

Review the literature and describe the complications associated with each of the anterior tibial tendon transfer (ATTT) techniques described.

**Methods:**

A systematic review of the literature was performed with the keywords ‘’clubfoot’’, ‘’Ponseti’’ and ‘’anterior tibial’’. Studies in patients with clubfoot recurrence, who underwent ATTT, whose method of tendon fixation was different from the classical method, were included.

**Results:**

Six studies were included in this systematic review, which described multiple techniques for tibialis anterior fixation: bone anchors, interference screws, endobotton, K-wires, transosseous suture, and suture to the plantar fascia. In the papers that described postoperative complications, no major complications were reported, however the samples are generally small.

**Conclusion:**

Several options have now emerged for tendon fixation in tendon transfers around the foot and ankle, including ATTT for treatment of relapsed clubfoot. To our knowledge this is the first paper that questioned the potential complications associated with the use of these new techniques. Due to the scarcity of published works in favor of other fixation methods, we believe that the traditional method is the optimal one for the transfer of the tendon of the tibialis anterior muscle.

**Supplementary Information:**

The online version contains supplementary material available at 10.1186/s12891-024-07621-9.

## Introduction

Clubfoot is one of the most frequent congenital musculoskeletal deformities, with an incidence of 1 to 7 per 1000 live births [1, 2]. The Ponseti technique has become the most widely accepted method of management of congenital clubfoot and has largely outdated traditional surgical management. Successful serial casting and meticulous bracing produce a well-corrected foot, requiring no invasive surgical intervention [2].

After complete correction with Ponseti method, 11 to 48% of patients tend to relapse and is usually a consequence of poor compliance with bracing [3]. Clubfeet have been explained by theories considering alterations in development, teratologic causes, intrauterus malpositioning, enviroment agents; none of these is strongly supported by literature. Despite this, relapse is a process caused by the same biologic mechanism that causes the deformity intrauterus [4]. Studies demonstrate differences in the posteromedial tissues in the leg and foot with connected tissue formed by wavy dense collagen and different cells, with some features similar to muscle tissue. The theory implicates possible “contractions” that drive the foot into the clubfoot position. This is more important when baby is born, and progressively milder as the child growths. The foot abduction brace is the most efficient tool to prevent relapses up to four years of age. Relapses are rare after 4 years and as the child grows, the foot can have difficulties to adapt to the stretching of soft tissues necessary to gain length [4]. This is the second obstacle to maintain correction, depending on how hypoplastic calf is and it is worst with growth spurts.

Relapses are detected with loss of dorsiflexion, and then a discrete equinus and varus deformity of the heel appears, frequently without an increase in the adductus and cavus of the forefoot [3, 4].

Anterior tibial tendon transfer (ATTT) is a widely advocated treatment for the treatment of clubfoot relapses, especially when performed in association with Ponseti’s method [5]. In the study by Zionts and co-workers the probability of undergoing an ATTT after the Ponseti method was 29% at age six [6]. In congenital clubfoot there is an imbalance between the inverters and everters of the foot, which contributes to the recurrence of the deformity [5]. Thus, the goal of ATTT is to restore this balance through the more lateral insertion of this tendon.

Different techniques of ATTT are described in the literature [7]. Classic ATTT technique described by Ponseti consists of detachment of the whole anterior tibial tendon, without opening extensor retinaculum, and reinserting it through a tunnel to the third cuneiform. Tendon fixation is performed by pull out technique with button at the plantar surface of the foot [8]. Sometimes, there is a pressure on the plantar surface of the foot, causing erhitema and some irritation. That happens when foot is not held in maximum dorsiflexion and abduction positioning after the transfer, and protected by positioning in the cast.

Multiple methods of anterior tibial tendon fixation after transfer have been described in the literature recently, including the use of anchors or interference screws [9–13]. However, little has been discussed about the potential complications associated with each of the techniques, such as loss of fixation or tension, infection, and skin bruises.

Thus, the purpose of this study is to review the literature and describe the complications associated with each of the techniques described.

## Methods

A systematic review of the literature was conducted. The keywords “clubfoot”, “Ponseti” and “anterior tibial” were used to search the PubMed, ScienceDirect, Scielo and Lilacs databases.

Inclusion criteria were patients with relapsing clubfoot who underwent ATTT, whose tendon fixation was different from the classic pull-out techniques described by Ponseti, in patients that were treated initially by Ponseti Method.

Papers where the method of fixation of the tibialis anterior tendon was not described and in which the procedure was performed for pathologies other than relapsed clubfoot were excluded.

Each potentially eligible article was reviewed, as were its references, and additional titles meeting the inclusion criteria articles were included.

Table 1 summarizes the quality of the selected studies using the methodological index for non-randomized studies (MINORS)*.

**Table 1:** Summary of the quality of the selected studies using the methodological index for non-randomized studies (MINORS)*

	Ayub et al. 2023 [13]	Yasin et al. 2020 [12]	Rhee et al. 2020 [11]	Pedraza et al. 2021 [14]	Mindler et al. 2020 [3]	Ploeger et al. 2022 [15]
A stated aim	2	2	2	2	2	2
Inclusion of consecutive patients	1	0	1	2	2	2
Prospective collection of data	2	1	2	2	2	1
Endpoints appropriate to the aim of the study	2	2	2	2	1	1
Unbiased assessment of the study endpoint	0	0	0	0	0	0
Follow-up period appropriate to the aim of the study	1	0	1	1	1	0
Loss of follow-up less than 5%	2	2	2	2	2	2
Prospective calculation of the study size	0	0	0	0	0	0
Total score**	11	7	10	11	10	8
Quality of the study***	low	low	low	low	low	low

*without additional criteria in the case of comparative studies, **record as 0 (non-reported), 1 (reported but inadequate), or 2 (reported and adequate), ***studies with a total score ≥ 12 were rated as having a high methodological quality

## Results

**Fig. 1:**
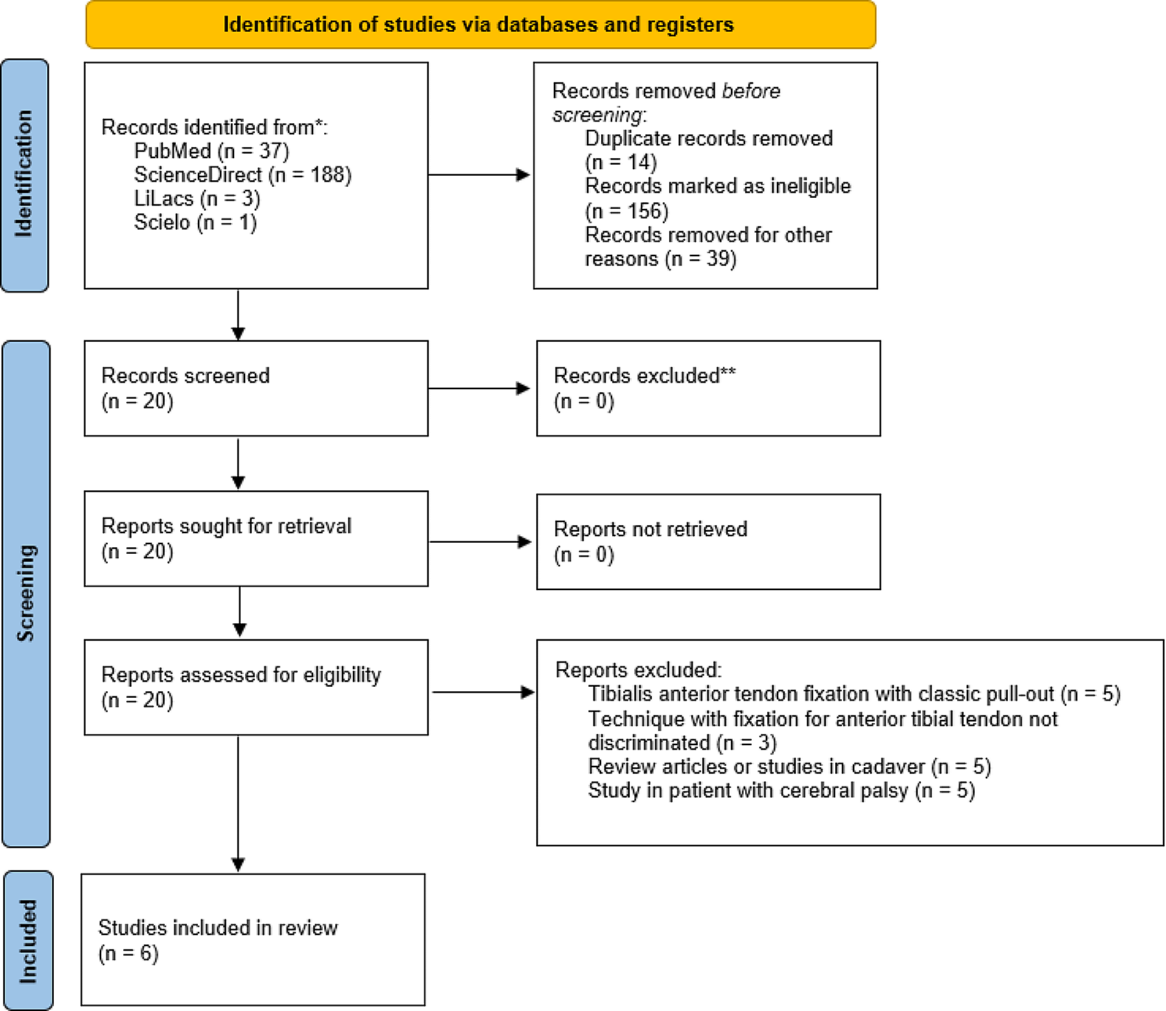
Article selection algorithm

The literature review with the keyword’s “clubfoot”, “Ponseti” and “anterior tibial” yielded 229 results in four databases: 37 results in PubMed, 188 in ScienceDirect, 3 in Lilacs and 1 in Scielo. After exclusion of duplicate articles and those irrelevant to the topic, the abstracts of the articles of interest were reviewed, as were their references, to identify additional articles (Fig. 1). Five articles met the inclusion criteria (Table 2). Were evaluated type of technique, material and complication after the procedure.

**Table 2:** Summary of the articles included in the systematic review

Reference		Treatment performed	Nr. of feet included	Fixation technique	Complications / Recurrences	
Ayub et al. 2023 [13]	ATTT ± other procedures (lengthening of the Achilles tendon and/or posteromedial release and/or osteotomy)		77 feet (56 idiopathic; 10 atypical/complex idiopathic; 11 syndromic)	Anchor	1 - recurrence of dynamic supination (insufficient tensioning) 1 - superficial infection without the need to remove the anchor 7 - need to repeat casts for recurrent loss of dorsiflexion and/or abduction 4 - reintervention (osteotomies or soft tissue release) to improve foot position (no cases of pullout of the anchor)	
Yasin et al. 2020 [12]	ATTT		26 feet	K-wire from lateral to medial, crossing the bone tunnel perpendicularly, crossing the prepared tendon with the suture	3 - minor skin erosion at the pin site with recovery after pin removal (none of them had a wound infection or tendon anchoring failure)	
Rhee et al. 2020 [11]	ATTT ± other procedures (lengthening of the Achilles tendon, posterior release and/or osteotomies)		34 feet	Endobutton	2 - recurrence treated conservatively 1 - loss of endobutton fixation 1 - infection requiring removal of the button 1 - pressure zone in the plant of the foot due to plaster	
Pedraza et al. 2021 [14]	ATTT ± achilles tendon tenotomy		54 feet Group 1–32 feet Group 2–14 feet	Group 1 - external button Group 2 - suture to plantar fascia	Group 1: 5 - superficial infection 1 - deep infection 14 - pain at the site of fixation	Group 2: 0 - superficial infection 0 - deep infection 4 - pain at the site of fixation
Mindler et al. 2020 [3]	ATTT ± achilles tendon lengthening ± percutaneous plantar fasciotomy		25 feet	Endobutton + biotenodesis screew		
Ploeger et al. 2022 [15]	ATTT ± other procedures (lengthening of the Achilles tendon, percutaneous release of the plantar fascia)		26 feet	Transosseous suture in lateral cuneiform	3 - relapse after 12 months of follow-up	

It was found that in some papers the anterior tibial tendon fixation technique was not described. Additionally, most of the papers with ATTT in the relapsed clubfoot use the classic pull-out technique, with an external button, first described by Ponseti [7, 16–19]. The prospective study by Mindler and colleagues aimed to study the biomechanics of the foot after ATTT in children with relapsed clubfoot [3]. The ATTT was performed in 25 feet. The tendon was fixed with an endobutton combined with a biotenodesis screw. Unfortunately, postoperative complications were not reported in this study, so no conclusions can be drawn about this form of fixation. However, it is important to highlight their conclusions regarding the improvement of gait and mobility of the foot after ATTT. Forefoot supination in relation to the hindfoot and tibia was reduced during swing and at initial contact, and heal showed less dynamic varus and adduction. In kinect there was na increase of the power o maximum ankle dorsiflexion.

Another one that refer to the type of fixation is the one proposed by Pedraza et al. Also Pedraza et al. applied this type of fixation in their alternative ATTT technique. They carried out a prospective, longitudinal, and observational study [14]. The patients were divided into two groups according to the type of distal fixation used: 20 patients (32 feet) with external plantar button fixation and 14 (22 feet) with suture to the plantar fascia. They reported significant differences according to the characteristics in superficial infection and pain in distal fixation after removing the cast, these being significantly higher in the group with plantar button. These differences probably explain why the group treated with suture to the plantar fascia had better tolerance to an early start of physical therapy after surgery.

Rhee et al. published their case series of 23 patients (34 feet) for ATTT using endobutton technique. They showed that they had a percentage of complications of 14.7% of which recurrence occurred bilaterally in 1 patient (5.9%) and there was a loss of fixation of the suture button (2.9%) in another case. Other complications included a cast-related pressure sore (2.9%) and an infection (2.9%) requiring irrigation with debridement along with hardware removal [11].

Yasin et al. described a different technique to avoid the skin complications related to the use of an external button for anterior tibial fixation. After making a bone tunnel in the lateral cuneiform and passing the anterior tibial tendon with the sutures, they used a K-wire from lateral to medial, crossing perpendicularly the bone tunnel and the anterior tibial tendon with the sutures, to anchor it after tensioning. This technique was used in 26 feet, and the authors report no cases of anterior tibial anchorage or tension loss or K-wire infection. Only in 3 cases there was slight skin erosions in the K-wire region, which resolved spontaneously within the first week after wire removal [12].

Also, Ploeger and his colleagues described a different technique of tibialis anterior tendon fixation for the treatment of clubfoot recurrence. These authors make a perforation of the lateral cuneiform, without reaching the plantar cortex, and anchor the tendon in that area, with a transosseous suture in the medial and lateral cortices, with the ends of the tendon preparation sutures knotting in the dorsal region of the lateral cuneiform. In this study 26 feet were included; no complications related to the fixation technique were reported. At 12 months follow-up 3 feet relapsed [15].

Recently, Ayub and colleagues published a series of patients who underwent ATTT with anchor tendon fixation. In this study, of the 77 feet submitted to ATTT, the authors report that there was no case of anchor failure; they had one case with recurrence of dynamic supination probably related to poor tendon tensioning; they also report one case of superficial infection, which was washed and debrided without the need to remove the anchor. Additionally, in 7 cases there was a need for repeat treatment with serial casts for recurrent loss of dorsiflexion and/or abduction, and in 4 cases a new surgical intervention was required to improve the position of the foot [13].

Those studies are all observacional and retrospective. 

## Discussion

Some authors are concerned about the classic fixation technique with an external button since skin perforation in the plantar region of the foot may be associated with infection or skin irritation associated with pressure from the button [11, 12, 20].

A recent study reported on a series of cases undergoing anterior or posterior tibial transfer with fixation by an external plantar button. Nine adult patients with multiple comorbidities (diabetes, history of amputation at different levels in the foot, history of chronic kidney disease or peripheral arterial disease) were included. No complications have been documented with this method of fixation. The authors state that this technique is not only safe for children, especially those with clubfoot, but is also safe for patients with a predisposition to skin complications (diabetes or peripheral arterial disease) [21]. This study corroborates the safety of this procedure.

Additionally, there is also some risk of injury to the plantar nerves and vessels when the surgeon performs the bone tunnel and the passage of the sutures from the tendon to the plantar region [10–14, 20]. The study by Radler et al. concludes that this technique is safe, and that nerve damage can be minimized if the drilling of the bone is directed towards the mid-region of the plantar surface; additionally, the use of a blunt needle to pass the sutures may be beneficial [22].

Another concern associated with the use of external button is the early failure of fixation associated with suture breakage or after removal of the button, since in this technique the maintenance of tension depends exclusively on the healing of the tendon to bone [10, 14, 23]. The strength of the fixation between surgery and during the post-operative immobilization and rehabilitation period is crucial to allow bone-tendon integration. Some studies have shown some vulnerability at the bone-tendon interface in the early stages of healing and suggest that this process requires 8 to 26 weeks [24]. Thus, there may be some advantage in using fixation methods that are maintained for longer periods of time, compared to the standard 6 weeks of external button fixation. However, there is already a lot of experience with this form of fixation, with very good long-term results reported in the literature and no reports of complications or failure [25].

Current publications report that there are complications associated with the use of new fixation devices in foot tendon transfers, namely interference screws. The only article that used an interference screw for tibialis anterior fixation and met the inclusion criteria for this review does not describe postoperative complications. Clanton et al. in their case series of 31 patients who underwent different tendon transfers in the foot, using bioabsorbable screws, demonstrated a complication rate of 39%. According to the authors, all the complications reported were most likely related to the tendon transfer procedure itself, and not directly related to the bioabsorbable screw [26]. There are several problems described in the literature with the use of this method of fixation, including screw breakage, screw loosening, inflammatory reactions, and tendon laceration during screw insertion [26]. Additionally, these materials are associated with high costs. Moreover, cuneiform ossification is a limitation in younger children [20].

Rhee and colleagues in their work using endobutton, concluded that using button suture in ATTT is a safer procedure, with theoretical advantage of providing stronger fixation and reducing the risk of skin pressure necrosis compared to the standard external button technique [11]. The results of this study are corroborated by the results of a biomechanical study in cadavers, where a comparison of traditional external button fixation and internal suspension was performed in ATTT. The results showed that the internal button fixation has significantly less displacement of the tendon within the bone tunnel than the external button technique, both with dynamic and static loading [10]. Nevertheless, it is not known how clinically significant this difference is and the price of these suspension systems are much higher compared to the traditional technique.

It is not a lesser fact than in a survey made to the members of the Pediatric Orthopedic Society of North America (POSNA), Hosseinzadeh et al. report that the preferred technique is a transfer of the tendon to the lateral cuneiform (73%), and the majority (72%) use a button as the method of fixation. However, it does not refer to which type of button [27].

In recent years the use of suture anchors has grown exponentially. Fennel et al. in a cadaveric study recommend suture anchors as they would be easier to insert and less traumatic to the bone than the bone tunnel technique [28]. However in vivo situation is very different because pull-out fixation also depends on integration of the tendon into the bone tunnel.

**Fig. 2:**
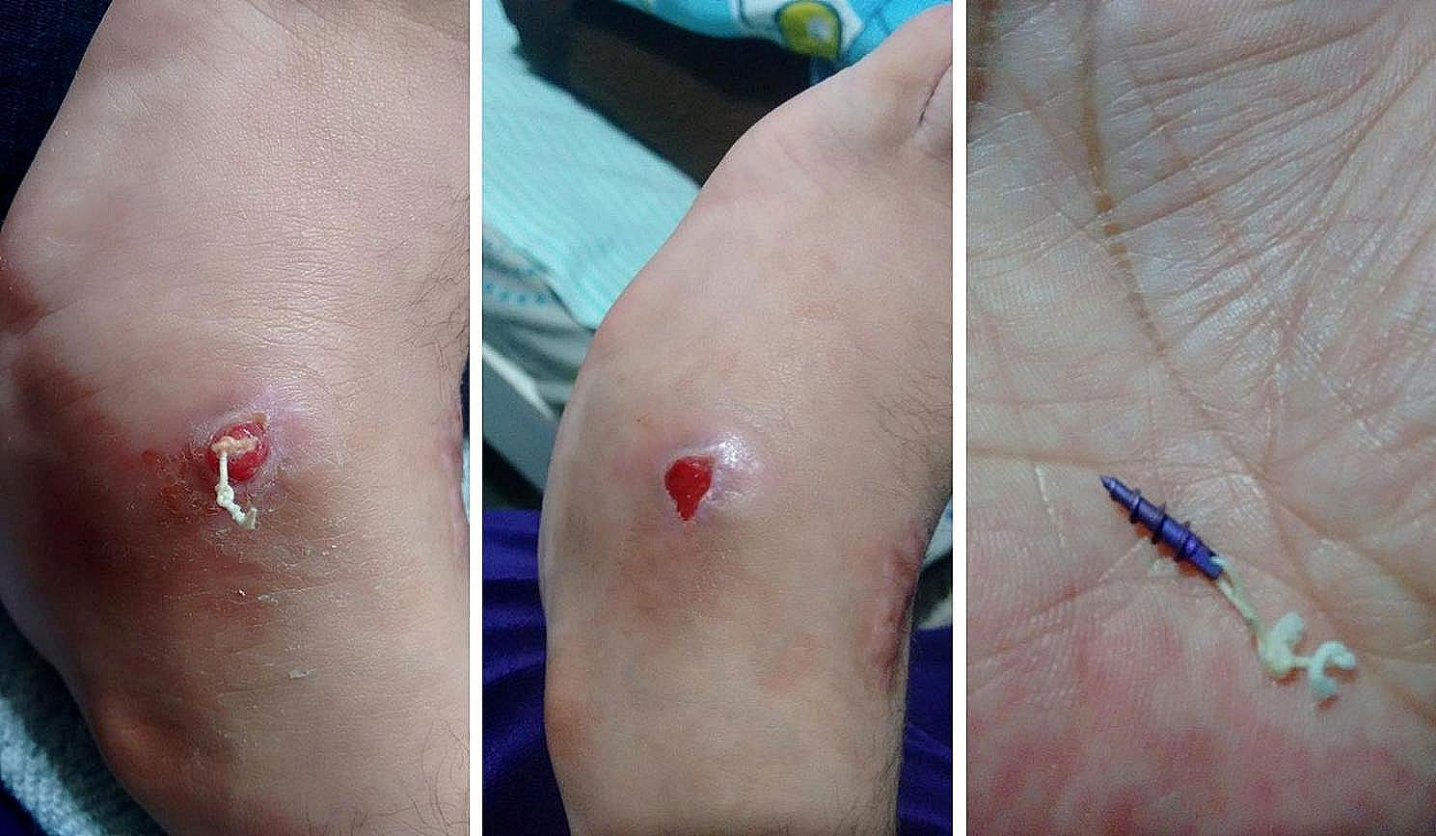
Loosening and infection of an anchor suture. Cortesy by Dr Rafael Batalha from Brazil

**Fig. 3:**
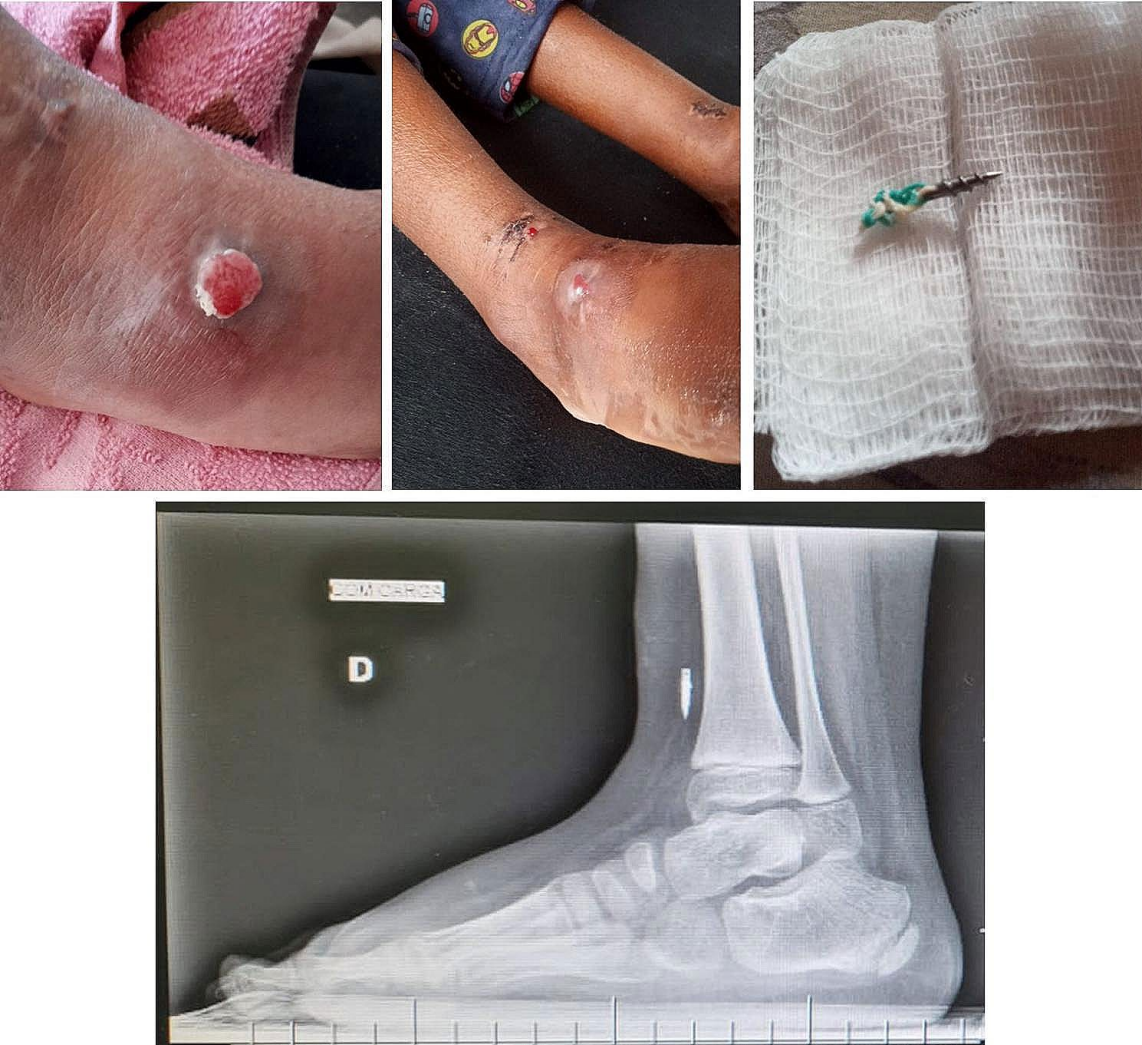
Loosening and exposition of the suture anchor. Cortesy of Dr Rodrigo Branco from Brazil

**Fig. 4:**
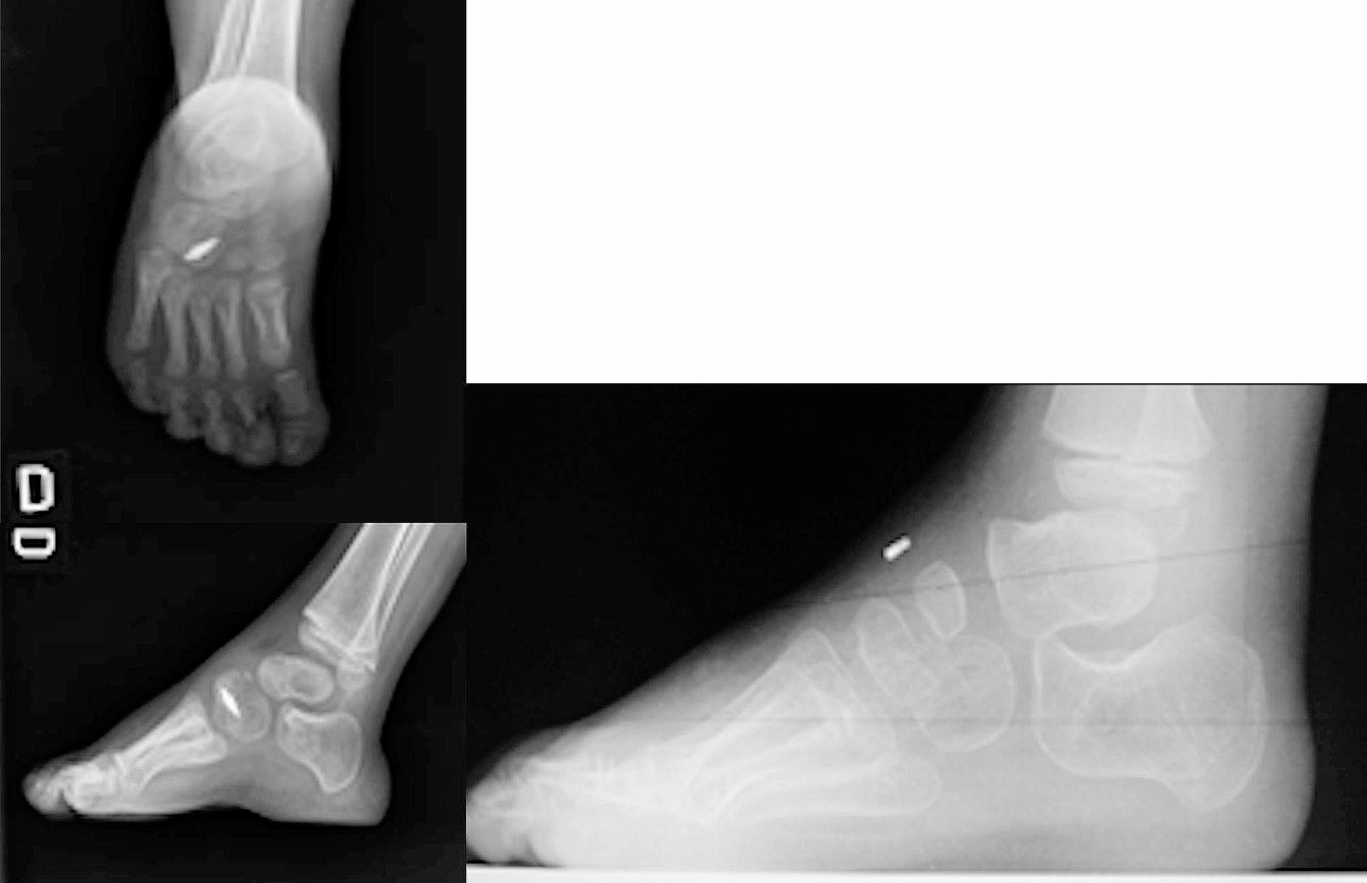
Immediate post-operative X-ray (left) and X-ray 4 months post-operative (right), demonstrating loosening of the bone anchor. Cortesy by Dr Cristina Alves from Portugal / Ponseti International Meeting Barcelona 2014

The only paper that was found that reported the use of anchors in ATTT only mentions one case in which the correction of dynamic supination was incomplete due to inadequate tendon tensioning, and they do not report other complications related to implant failure [13]. However, we have case reports of complications by different experienced surgeons. According to their experience, they did not have optimal results. They also required to perform revision surgery due to loosening of the anchor. The photos are shown in Figs. 2 and 3, and 4.

Other tendon fixation techniques have also been described, either with K-wires [12] or with transosseous suture [15]. In these studies, no complications related to the type of fixation were described, and they do not require specific material that increases the cost of the procedure. Still, they are small case series, so their effectiveness and safety cannot be extrapolated.

The classic technique of anterior tibial fixation with pull-out and external button is inexpensive, technically simple, and effective, as shown in several studies [7, 16, 18, 21, 25].

Gray and colleagues, in a prospective study, compared a group with relapsed clubfoot after treatment with Ponseti’s method, with an indication for ATTT (24 feet), with a group of patients with the same initial treatment but who had no relapse (18 feet). They concluded that ATTT is an effective procedure, which restores the balance of eversion and reversal force. Additionally, they found that this procedure results in similar function and satisfaction as children with clubfoot treated with Ponseti’s method who did not relapse. In this study, the anterior tibial tendon was fixed in the lateral cuneiform with the classic technique over a plantar external button and no postoperative complications were reported [16].

Agarwal et al. compared three different techniques (ATTT for third cuneiform – classic Ponseti technique, ATTT for the cuboid, and split transfers to cuboid) and found no statistically significant differences in foot and ankle function between the different techniques, however the technique described by Ponseti tended to show better dorsiflexion and eversion compared to other techniques [7]. In all techniques the tendon was fixed with the classic pull-out technique and the authors report that no major complications were observed in any of the groups [7].

Also, Thompson et al., retrospectively evaluated 137 feet undergoing ATTT for clubfoot recurrence. The plantar pull-out technique was used for fixation of the tibialis anterior. The authors denied postoperative complications, including loss of tendon tension, postoperative infections, or neurovascular damage [17].

In another study, two groups undergoing ATTT for clubfoot relapse (35 feet) were compared: one group treated initially with manipulations according to Ponseti’s technique and another group treated initially with posteromedial release [18].

The percentage of relapse was similar in both groups, reinforcing the fact that posteromedial release does not reduce the incidence of relapse. On the other hand, relapses in feet initially treated with extensive posteromedial release had higher stiffness when compared to those treated with Ponseti’s method. The ATTT was performed with the classic pull-out technique and there is no reference to postoperative complications related to the method of tendon fixation. The clinical outcomes assessed at the end of skeletal maturity were significantly different in the 2 series of patients, with better clinical outcomes in the group of patients initially treated with Ponseti’s method, this difference probably being related to the increased foot stiffness in the group of patients initially undergoing extensive posteromedial releases [18].

Holt et al. demonstrated that the treatment algorithm based on serial casts to regain correction, new Achilles tenotomy and ATTT is effective in the long term [25]. They demonstrated that ATTT improves foot function in adult patients who had been treated for recurrence of idiopathic clubfoot during childhood, with a follow-up of 37 to 55 years. They used the traditional fixation method, which confirms the effectiveness and safety of this technique.

Masrouha and Morcuende studied relapses after ATTT, a retrospective study in which they observed that of 66 patients, ten had recurrence after ATTT [19]. In all of them they used the traditional fixation method. The authors conclude that the recurrence would be related to performing the ATTT at an early age or neurologic deficits and not necessarily due to the fixation method used.

## Conclusion

Several options have now emerged for tendon fixation in tendon transfers around the foot and ankle, including ATTT for treatment of relapsed clubfoot. To our knowledge this is the first paper that questioned the potential complications associated with the use of these new techniques, such as loss of fixation or tension, infection, and skin bruises. They are severe because compromise the results of surgery. Due to the scarcity of published works in favor of other fixation methods, we believe that the traditional method is the optimal one for the transfer of the tendon of the tibialis anterior muscle.

### Electronic supplementary material

Below is the link to the electronic supplementary material.

Supplementary Material 1

## Data Availability

The datasets used and/or analyzed during the current study are available from the corresponding author on reasonable request.
